# Genetic diversity and population differentiation in *Earliella scabrosa*, a pantropical species of Polyporales

**DOI:** 10.1038/s41598-023-50398-5

**Published:** 2023-12-27

**Authors:** Boris Armel Olou, Apollon D. M. T. Hègbè, Meike Piepenbring, Nourou Soulemane Yorou

**Affiliations:** 1 Research Unit Tropical Mycology and Plant-Soil Fungi Interactions (MyTIPS), Faculty of Agronomy, University of Parakou, BP 123 Parakou, Benin; 2 Mycology Research Group, Faculty of Biological Sciences, Goethe University Frankfurt am Main, Biologicum, Max-von-Laue-Str. 13, 60438 Frankfurt am Main, Germany

**Keywords:** Fungi, Fungal genetics

## Abstract

*Earliella scabrosa* is a pantropical species of Polyporales (Basidiomycota) and well-studied concerning its morphology and taxonomy. However, its pantropical intraspecific genetic diversity and population differentiation is unknown. We initiated this study to better understand the genetic variation within *E*. *scabrosa* and to test if cryptic species are present. Sequences of three DNA regions, the nuclear ribosomal internal transcribed spacer (ITS), the large subunit ribosomal DNA (LSU), and the translation elongation factor (EF1α) were analysed for 66 samples from 15 geographical locations. We found a high level of genetic diversity (haplotype diversity, Hd = 0.88) and low nucleotide diversity (π = 0.006) across the known geographical range of *E*. *scabrosa* based on ITS sequences. The analysis of molecular variance (AMOVA) indicates that the genetic variability is mainly found among geographical populations. The results of Mantel tests confirmed that the genetic distance among populations of *E. scabrosa* is positively correlated with the geographical distance, which indicates that geographical isolation is an important factor for the observed genetic differentiation. Based on phylogenetic analyses of combined dataset ITS-LSU-EF1α, the low intraspecific divergences (0–0.3%), and the Automated Barcode Gap Discovery (ABGD) analysis, *E. scabrosa* can be considered as a single species with five different geographical populations. Each population might be in the process of allopatric divergence and in the long-term they may evolve and become distinct species.

## Introduction

Data on genetic variation within a population is useful to discuss species concepts, cryptic species, breeding patterns, degree of relatedness, differentiation, and gene pool disruptions^1–4^. Low levels of genetic diversity may reduce the ability of populations to cope with environmental changes and other threats, leading species to become endangered or even extinct^5–7^. High genetic diversity allows species to adapt to environmental changes^8^. It is therefore essential to understand the genetic diversity and population structure of a species. This is important in particular for species with a wide geographical distribution as these may include multiple genetic lineages or cryptic species^9,10^.

*Earliella* Murrill (Basidiomycota, Polyporales) includes a single species *Earliella scabrosa* (Pers.) Gilb. & Ryvarden. The species was described as *Polyporus scabrosus* Pers. on the basis of a specimen collected in the Marianas islands (exact position not known)—about 1500 km east of the Philippine islands (south of Japan) in the Pacific Ocean. *Earliella scabrosa* is a saprotrophic species that colonises dead wood. It is also reported to be an opportunistic pathogen of plants and humans. As a human pathogen, it can infect and cause endophthalmitis and cutaneous fungal septic emboli^11,12^. *Earliella scabrosa* is also recognized as a medicinal species with anticancer, antifungal and antimicrobial activities as well as wound healing capabilities^13–15^. The species produces enzymes for the biotransformation of dyes in solid state fermentation and for the removal of bromocresol green, thus purifying water^16,17^.

**Figure 1:**
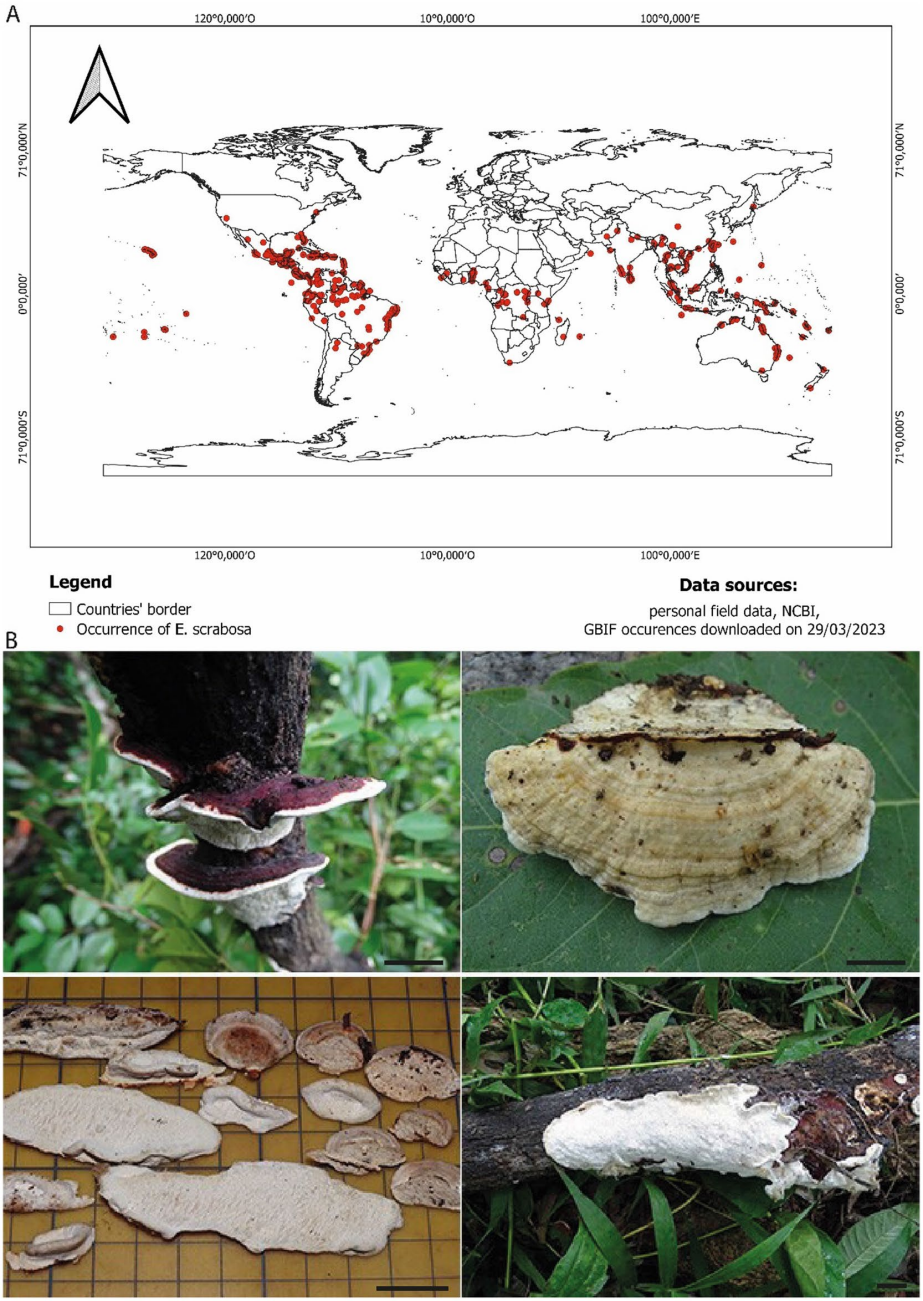
(**A**) Distribution map of *Earliella scabrosa*. Occurrences of all collected specimens during our mycological surveys as well as other occurrences available on the GBIF (Global Biodiversity Information Facility) website and those of the sequences obtained from the NCBI website were projected onto the map using the QGIS v3.28.0. (**B**) Morphological variability of basidiomata of *Earliella scabrosa*. Scale bar 2 cm.

Morphologically, *E. scabrosa* can be easily recognized due to its effuse-reflexed basidiomata with reddish cuticle and irregular, elongated or sinuous pores^18^. It is a common fungal species distributed all over the tropical regions of both hemispheres (Fig. 1A). Although the shape and colour of the basidiomata vary considerably (Fig. 1b), *E*. *scabrosa* is considered as a single species. Information on intraspecific diversity and genetic variability within and among populations is not available.

Studies on many fungi previously thought to belong to a single species like *Cryptococcus neoformans* (San Felice) Vuill.^19,20^, *Paxillus involutus* (Batsch) Fr.^21^, *Serpula himantioides* (Fr.) P. Karst.^22^, and *Trametes elegans* (Spreng.) Fr.^23,24^ have been identified as species complexes that each contain two or more cryptic species. The above-mentioned consideration could also account for *E*. *scabrosa* with a wide distribution (pantropical), but, until now, we have no information on the genetic diversity within this species.

The most widely used approaches to assess genetic diversity from DNA sequences are nucleotide diversity and haplotype diversity^25–27^. Several highly variable DNA molecular markers are used for intra- and inter-specific genetic characterisation in plants, animals, and fungi^28–31^. In fungi, the internal transcribed spacer (ITS) region of nuclear ribosomal DNA has been most intensively sequenced and used^32–35^. It has been widely used for species identification, phylogenetic analyses, and the investigation of genetic variability^36–40^. In general, the fungal ITS marker provides higher interspecific resolution, with some degree of intraspecific variability^34,36^.

The goal of this study is to examine the extent of genetic divergence within *E*. *scabrosa* throughout most its geographical range and discuss these results in light of the potential existence of cryptic species within this taxon. At present, there is no DNA sequence for African specimens of *E*. *scabrosa* in any public database. Therefore, we focused our attention on African specimens in order to answer the following questions: (i) Is there genetic variation among individuals of *E*. *scabrosa* from the same geographical area? (ii) Is there genetic differentiation between populations in different geographic areas? (iii) If so, is the level of genetic differentiation positively correlated with geographic distances? (iv) Do the different populations of *E*. *scabrosa* form a single species or does this species contain cryptic (hidden) species?

## Materials and methods

### Specimen assembly

A total of 20 specimens of *E. scabrosa* were collected in Benin, Congo, and Guinea. Specimens were photographed in their natural environment before recording using a Sony camera, model DSC-HX400V. The geographic coordinates of occurrence of each specimen were recorded (Fig. 1). Small pieces of fresh basidiomata were placed in plastic bags half-filled with silica gel for gentle drying. The rest of the basidiomata were air- or oven-dried at 45–50 °C. Specimens are deposited at the mycological herbarium of the University of Parakou (UNIPAR) in Benin. For specimen data, see Supplementary S1.

### DNA extraction, amplification, sequencing, and genetic distances analysis

#### DNA extraction, amplification, and sequencing

Genomic DNA from dried specimen was extracted using the microwave method^41^ or Analytik Jena kit when the first technic did not yield good results. The extracted genomic DNA was amplified targeting two nuclear ribosomal DNA regions, the internal transcribed spacer region of rDNA (ITS) with the primer pair ITS-1F/ITS4^42,43^ and the D1–D4 domain of large subunit (LSU; 28S rDNA) with the primers LR0R/LR5^44^. Additionally, the protein-coding gene translation elongation factor 1-alpha (EF1α) was amplified using the primers EF1-983/EF1-2218^45^. The polymerase chain reaction (PCR) procedure for ITS was as follows: initial denaturation at 95 °C for 3 min, followed by 35 cycles at 95 °C for 30 s, 52 °C for 30 s and 68 °C for 1 min, and a final extension at 68 °C for 3 min. The PCR procedure to amplify the LSU rDNA sequence differed from the one for the ITS rDNA only by the annealing temperature (55 °C instead of 52 °C) and an increased cycle extension time (90 s per cycle). To amplify the protein-coding gene EF1α, the touchdown PCR protocol following Justo and Hibbett^46^ was used. The PCR products were further cleaned with QIAquick PCR Purification Kit according to the manufacturer’s instructions (QIAGEN GmbH, Hilden, Germany) and then sequenced at the company Eurofins Genomics Germany GmbH (https://www.eurofinsgenomics.eu/). All sequences used in this study are listed in Table 1 and the newly generated sequences deposited in GenBank.

**Table 1:** Species names, sample data, and GenBank accession numbers of sequences of *Earliella scabrosa* obtained in the context of the present study or retrieved from GenBank.

Species name	Voucher or strain	Origin	GenBank N°			References
			ITS	LSU	EF1α	
*Daedalea quercina*	FP56429	USA	KY948809	KY948883		^57^
*Earliella scabrosa*	117_01_01	Sri Lanka	MT507861			^58^
*Earliella scabrosa*	216-TWTDW2	China	KU194310			^58^
*Earliella scabrosa*	biocode08-30	French Polynesia	MZ996924			^59^
*Earliella scabrosa*	biocode08-59	French Polynesia	MZ996933			^59^
*Earliella scabrosa*	biocode08-82	French Polynesia	MZ996931			^59^
*Earliella scabrosa*	biocode08-96	French Polynesia	MZ996935			^59^
*Earliella scabrosa*	biocode08-110	French Polynesia	MZ996936			^59^
*Earliella scabrosa*	biocode08-145	French Polynesia	MZ996925			^59^
*Earliella scabrosa*	biocode08-158	French Polynesia	MZ996928			^59^
*Earliella scabrosa*	biocode08-183	French Polynesia	MZ996926			^59^
*Earliella scabrosa*	biocode09-248	French Polynesia	MZ996930			^59^
*Earliella scabrosa*	biocode09-324	French Polynesia	MZ996927			^59^
*Earliella scabrosa*	biocode09-337	French Polynesia	MZ996929			^59^
*Earliella scabrosa*	biocode09-544	French Polynesia	MZ996932			^59^
*Earliella scabrosa*	BRFM1106	French Guiana	JX082364			^58^
*Earliella scabrosa*	CIRM-BRFM 1817	USA	OL685338			^60^
*Earliella scabrosa*	CLZhao 3722	China	MK268896			^58^
*Earliella scabrosa*	CLZhao 3730	China	MK268897			^58^
*Earliella scabrosa*	CLZhao 3989	China	MH114644			^58^
*Earliella scabrosa*	CLZhao 4008	China	MH114645			^58^
*Earliella scabrosa*	CR45	Venezuela	JN164992			^46^
*Earliella scabrosa*	CR95	Venezuela	JN165008			^46^
*Earliella scabrosa*	CrIre	Venezuela	JN165006			^46^
*Earliella scabrosa*	Cui 6236	China	KC867366	KC867485	KX838431	^58^
*Earliella scabrosa*	extr19	Taiwan	MH605432			^58^
*Earliella scabrosa*	FBP11	Viet Nam	MF521432			^58^
*Earliella scabrosa*	FBP13	Viet Nam	MF521433			^58^
*Earliella scabrosa*	FDNa20	Viet Nam	MF521434			^58^
*Earliella scabrosa*	FLAS-F-61025	USA	MH211697			^58^
*Earliella scabrosa*	He31	China	KC867365	KC867484		^58^
*Earliella scabrosa*	KP1	Thailand	KF860879			^58^
*Earliella scabrosa*	MH_60	Pakistan	MN892530			^58^
*Earliella scabrosa*	NTOU5409	Taiwan	MN592930			^61^
*Earliella scabrosa*	NTOU5411	Taiwan	MN534946			^61^
*Earliella scabrosa*	NTOU5413	Taiwan	MN534947			^61^
*Earliella scabrosa*	NTOU5416	Taiwan	MN534948			^61^
*Earliella scabrosa*	NTOU5882	Taiwan	MW940757	MW881468		^58^
*Earliella scabrosa*	NTOU5883	Taiwan	MW940758	MW881469		^58^
*Earliella scabrosa*	NTOU5884	Taiwan	MW940759	MW881470		^58^
*Earliella scabrosa*	PR1209	Puerto Rico	JN165009			^58^
*Earliella scabrosa*	UOC-BIB-MB03	Sri Lanka	KP734204			^58^
*Earliella scabrosa*	UOC DAMIA D13b	Sri Lanka	KR706165			^58^
*Earliella scabrosa*	UOC DAMIA D37	Sri Lanka	KR706167			^58^
*Earliella scabrosa*	URM7788	Brazil	MG870412			^62^
*Earliella scabrosa*	ZD16091703	China	MN523328			^58^
*Earliella scabrosa*	ZD16091705	China	MN523327			^58^
*Earliella scabrosa*	OAB0062	Benin	ON876009			This study
*Earliella scabrosa*	OAB0067	Benin	OR116224			This study
*Earliella scabrosa*	OAB0119	Benin	OR116225	OR116239	OR148937	This study
*Earliella scabrosa*	OAB0186	Benin	OR116226			This study
*Earliella scabrosa*	OAB0212	Benin	OR116227	OR116240	OR148938	This study
*Earliella scabrosa*	OAB0285	Benin	OR116228	OR116241	OR148939	This study
*Earliella scabrosa*	OAB0286	Benin	OR116229			This study
*Earliella scabrosa*	OAB0626	Benin	OR116230			This study
*Earliella scabrosa*	OAB0630	Benin	OR116231	OR116242	OR148940	This study
*Earliella scabrosa*	OAB0691	Benin	OR116217			This study
*Earliella scabrosa*	OAB0729	Benin	OR116218			This study
*Earliella scabrosa*	OAB1021	Guinea	OR116219			This study
*Earliella scabrosa*	OAB1024	Guinea	OR116220			This study
*Earliella scabrosa*	OAB1025	Guinea	OR116221			This study
*Earliella scabrosa*	OAB1027	Guinea	OR116234			This study
*Earliella scabrosa*	OAB1030	Guinea	OR116222			This study
*Earliella scabrosa*	OAB0755	Benin	OR116232			This study
*Earliella scabrosa*	OAB0845	Benin	OR116233			This study
*Earliella scabrosa*	OAB1120	Benin	OR116235			This study
*Earliella scabrosa*	ALN050	Congo	OR116223			This study
*Trametes suaveolens*	FP102529sp	USA	JN164966	JN164807	JN164890	^46^

#### Checking the affiliation of the name *Earliella scabrosa* applied to sequences in GenBank

On July 16th, 2022, all sequences of the ITS region labelled as *E. scabrosa* in GenBank (n = 76) were downloaded. The 76 ITS sequences of *E*. *scabrosa* from GenBank were aligned with 20 ITS sequences of *E. scabrosa* newly generated in this study. Sequences of the type species of the genera *Daedalea* and *Trametes* are added as outgroup. A maximum likelihood analysis was performed using IQ-tree 1.6.12 (http://www.iqtree.org/). Sequences of specimens from Mariana islands (type locality of *E*. *scabrosa*) are not available. However, all sequences (n = 66) of *E*. *scabrosa* that cluster together to form a single clade with sequences of specimens from other Pacific Ocean islands like French Polynesia are retained as *E*. *scabrosa* and were used for further analysis. Twenty-three sequences that were labelled *E*. *scabrosa* in GenBank did not form part of this clade. They are considered misidentifications and were not used for the present analysis (Supplementary S2).

#### Genetic diversity and relationships between geographic populations of *Earliella scabrosa*

A total of 66 ITS sequences belonging to *E. scabrosa* and annotated with data on geographic origin were used for the genetic analyses. These sequences were aligned and the resulting alignment was manually adjusted using Aliview. After this, the molecular diversity indices, such as the number of haplotypes (Nh), haplotype diversity (Hd), and nucleotide diversity (π) were estimated using *pegas* package^47^. To detect genetic differentiation within and among populations of *E*. *scabrosa*, an analysis of molecular variance (AMOVA) was performed using the alignment of 66 ITS sequences from 15 countries. The correlation between genetic and geographic distances was evaluated using a Mantel test^48^ and a linear regression. The phylogenetic relationships among the detected haplotypes are presented in a haplotype network. Statistical analyses have been implemented in the Integrated Development Environment RStudio (RStudio Team, 2021) for R software v4.1.2 (R Core Team, 2021).

#### Species delimitation within *Earliella scabrosa*

The same alignment with 66 sequences used in previous analyses is used here for species delimitation using the Automated Barcode Gap Discovery (ABGD) analysis^49^. The ABGD is a model-based method that delimit partitions of taxa, which can be recognized as species entities^49^. It sorts the sequences into hypothetical species based on the barcode gap. The ABGD analysis was performed using the Jukes-Cantor (JC69) distance with the relative gap width set to 1.0 and all other parameters were kept in default mode.

#### Phylogenetic relationship analyses

For phylogenetic analyses, sequences of ITS, LSU, and EF1α were used. Sequences of *Daedalea quercina* (L.) Pers. and *Trametes suaveolens* (L.) Fr. were used as outgroup. The sequences were aligned separately for each marker using the online mode of MAFFT version 7^50^. The resulting multiple sequence alignments were checked in Geneious 5.6.7 (Kearse et al*.*^51^, https://www.geneious.com), where the ends rich in gaps were manually trimmed. Further, the multiple sequence alignments were viewed and some bases were manually corrected using AliView^52^. The combination of ITS, LSU and EF1α alignments was used for phylogenetic relationship analyses. The best-fit evolutionary model was estimated for each region using ModelFinder implemented in IQ-tree 1.6.12 http://www.iqtree.org/^53^. Based on estimated evolutionary models, the phylogenetic tree inference of Maximum likelihood (ML) and Bayesian Inference (BI) were performed to verify the phylogenetic relationship between all sequences of *E. scabrosa*. The Maximum likelihood analysis was performed with Ultrafast Bootstrap (UFBoot**)**^54^ and the branch support was evaluated with 5000 replicates using the IQ-tree 1.6.12. The BI was executed using MrBayes v. 3.2.7 in command line mode (https://github.com/NBISweden/MrBayes) for five million generations until the standard deviation of split frequencies reached 0.01. Chain convergence was determined using Tracer v. 1.7.1 (http://tree.bio.ed.ac.uk/software/tracer/) and the first 25% (5000) of the trees were discarded as burn-in. The remaining trees were used to build the consensus tree using the Phylogenetic Tree Summarization (SumTrees) program within DendroPy v. 4.3.0., https://github.com/jeetsukumaran/DendroPy^55^. The topology of species resulting from ML is used, and to add the posterior probabilities (PP) of BI on the ML tree, the Phylogenetic Tree Summarization (SumTrees) program within DendroPy v. 4.3.0., was used. Then, the UFBoot values were added to the ML best tree that already having the posterior probabilities using IQ-tree^56^. The resulting tree is presented below in the Fig. 5 and the support values of UFBoot/PP are indicated on each node.

## Results

### Newly generated sequences

A total of 28 new sequences were generated, namely 20 ITS sequences, four LSU sequences, and four EF1α sequences. Sequences have been generated for the first time for specimens from Africa.

### Genetic diversity analysis

#### Haplotype diversity and distribution

**Figure 2:**
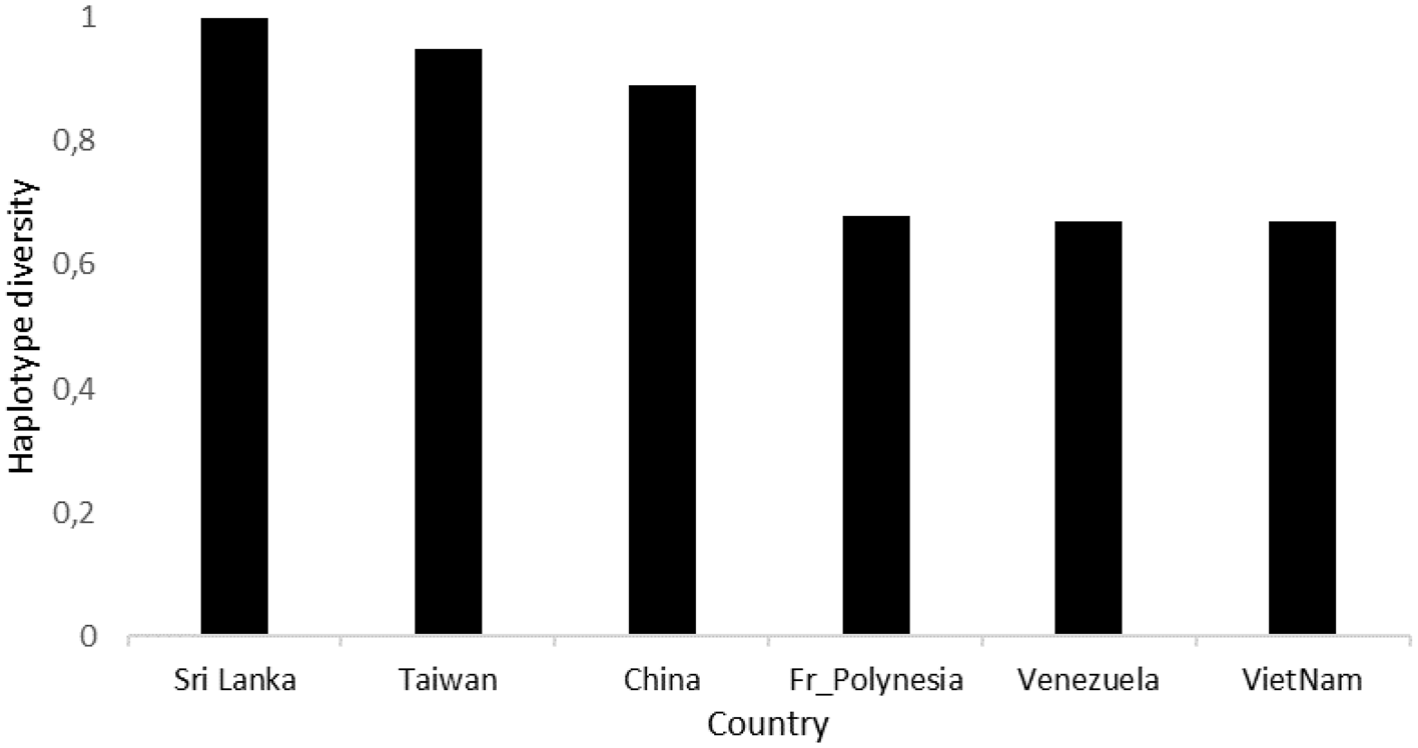
Haplotype diversity in countries with high scores.

The analysis of 66 ITS sequences of *E. scabrosa* with information on geographic origin revealed 24 haplotypes. The probability that two randomly selected alleles are different (haplotype diversity) is 0.880. The nucleotide diversity, which is the mean difference in nucleotide per base position when comparing DNA sequences pairwisely, is π = 0.0058. The highest number of haplotypes (Nh = 6) is recorded for China, French Polynesia and Taiwan while the highest haplotype diversity (haplotypes diversity = 1) is discovered for Sri Lanka (Fig. 2).

**Figure 3:**
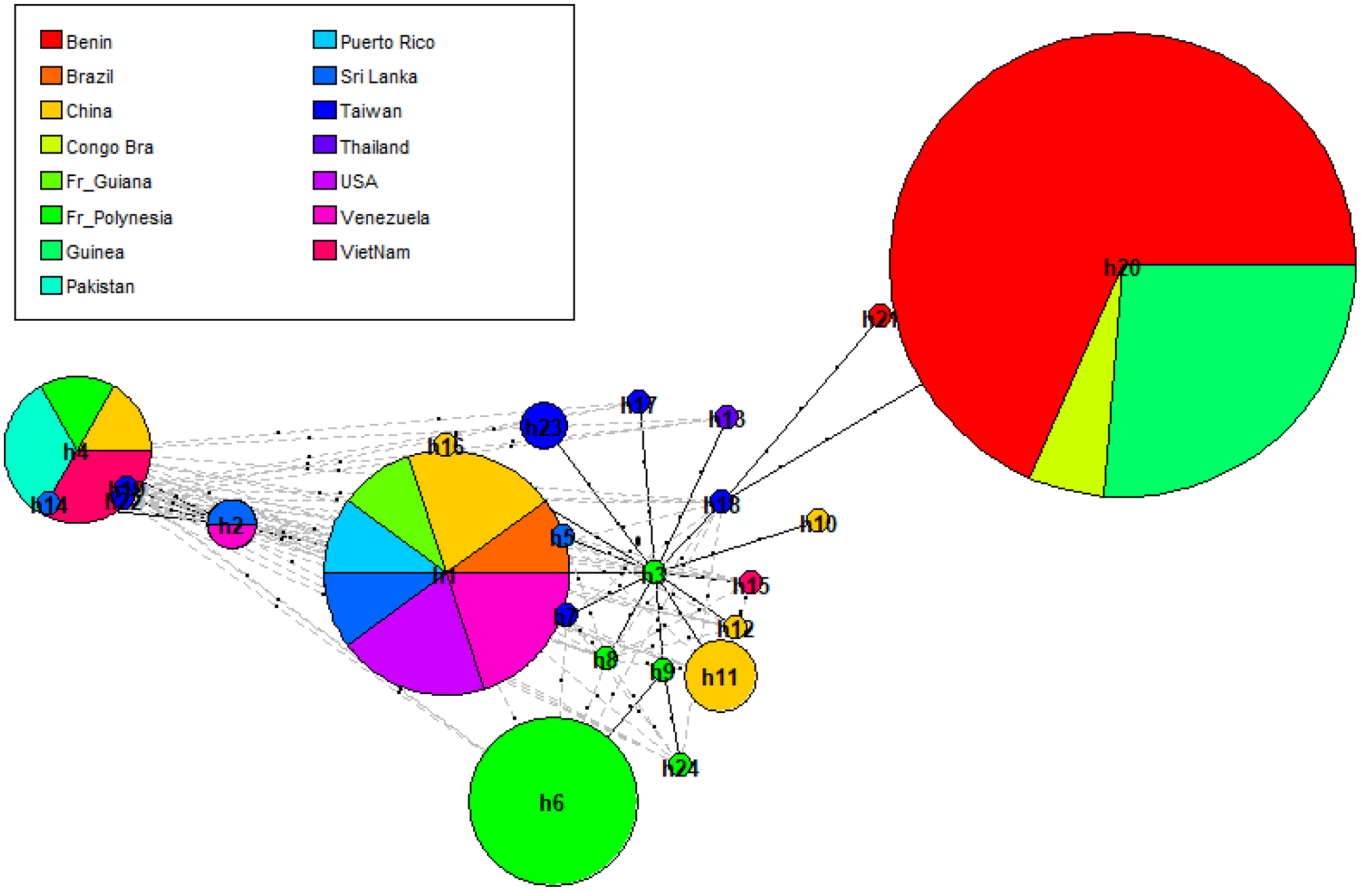
Haplotype network based on ITS sequences of *Earliella scabrosa*, including information on the geographical origin of the sequences by different colours. The circles represent different haplotypes. The sizes of the circles as well as the sizes of the pies reflect the frequency of detection of each haplotype. The lines connecting haplotypes represent their genetic distance and the short perpendicular lines indicate the number of nucleotide differences.

The haplotype distribution in all populations is shown in Fig. 3 and suggests a limited gene flow between populations between geographic areas. Of the 24 haplotypes in total, only four haplotypes namely h1, h2, h4, and h20 occur in several populations in different areas. Haplotype 20 (h20) is only distributed in three African countries, namely Benin, Congo Republic, and Guinea. Almost all haplotypes are linked to h3 which is from French Polynesia (Fig. 3). Our haplotype analyses revealed a high number of unique haplotypes mostly found in Taiwan and French Polynesia. The most common haplotypes are h20, h1, h6 and h4 which correspond to respectively 29%, 15%, 11%, and 9% of the individual DNA sequences included in the analysis.

#### Population structure and genetic differentiation

**Figure 4:**
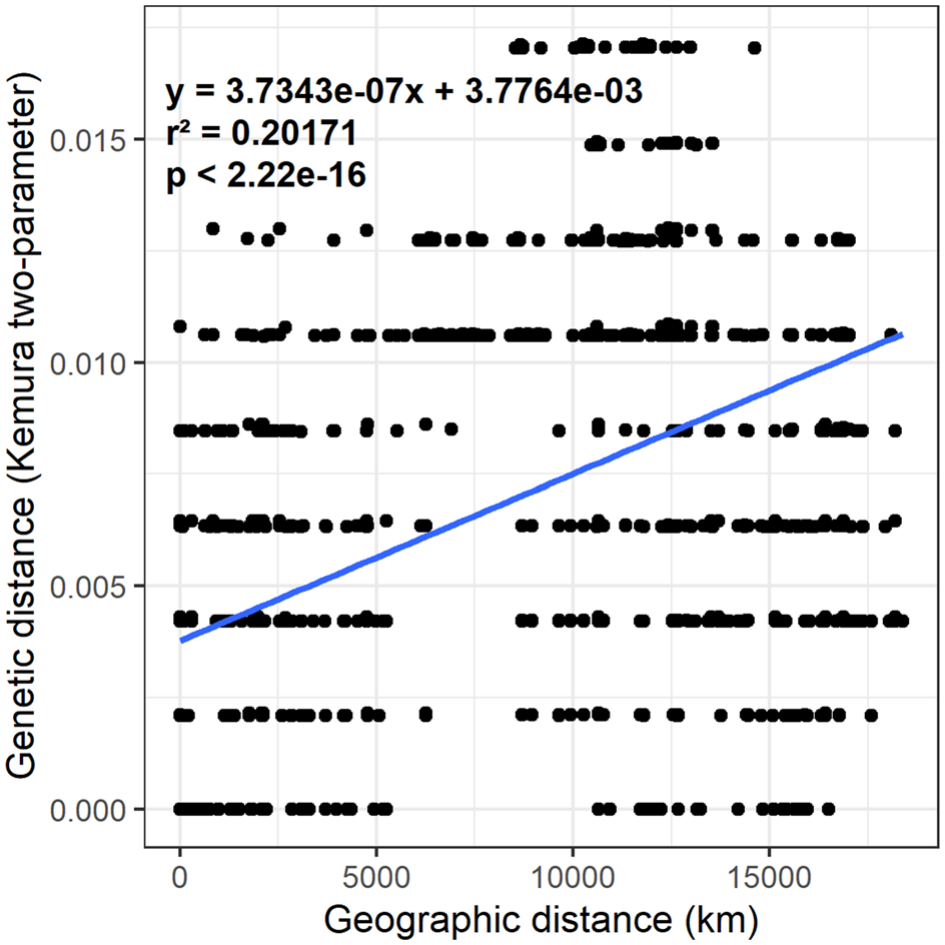
Influence of geographic distance on genetic distance in *Earliella scabrosa* populations.

The results from the analysis of molecular variance (AMOVA) showed significant genetic differentiation between the sequences of *E*. *scabrosa* from the source countries (ΦST = 0.82, *p* = 0, Table 2). The analysis suggests that nearly 82% of the genetic variation is explained by between country differences while only 18% of the genetic variation is explained by within country differences (Table 2). The result of the Mantel test shows a significant correlation between geographical distance and genetic distance. This is confirmed by the linear regression fit which indicates a positive correlation of the genetic distance and the geographic distance (Fig. 4).

**Table 2:** Molecular variance of *Earliella scabrosa* in different countries calculated by AMOVA.

Source of variation	df	Sum of squares	Variance component	% Variation	ΦST	*p*-value
Between country	14	0.0020423	1.459e−04	81.65	0.81652751	0
Within country	51	0.0003852	7.554e−06	18.35		
Total	65	0.0024275	4.117e−05

#### Species delimitation

The ABGD partitioned the ITS dataset into three initial partitions. The first two partitions with an intraspecific divergence of 0–0.2% divided sequences of *E*. *scabrosa* into 18 groups with each group representing a hypothetical species. All sequences from African specimens clustered together and formed a single hypothetical species (Supplementary S3). However, with an intraspecific divergence of only 0.3%, all sequences of *E*. *scabrosa* should be considered as one single species (Supplementary S4).

#### Phylogenetic analysis

**Figure 5:**
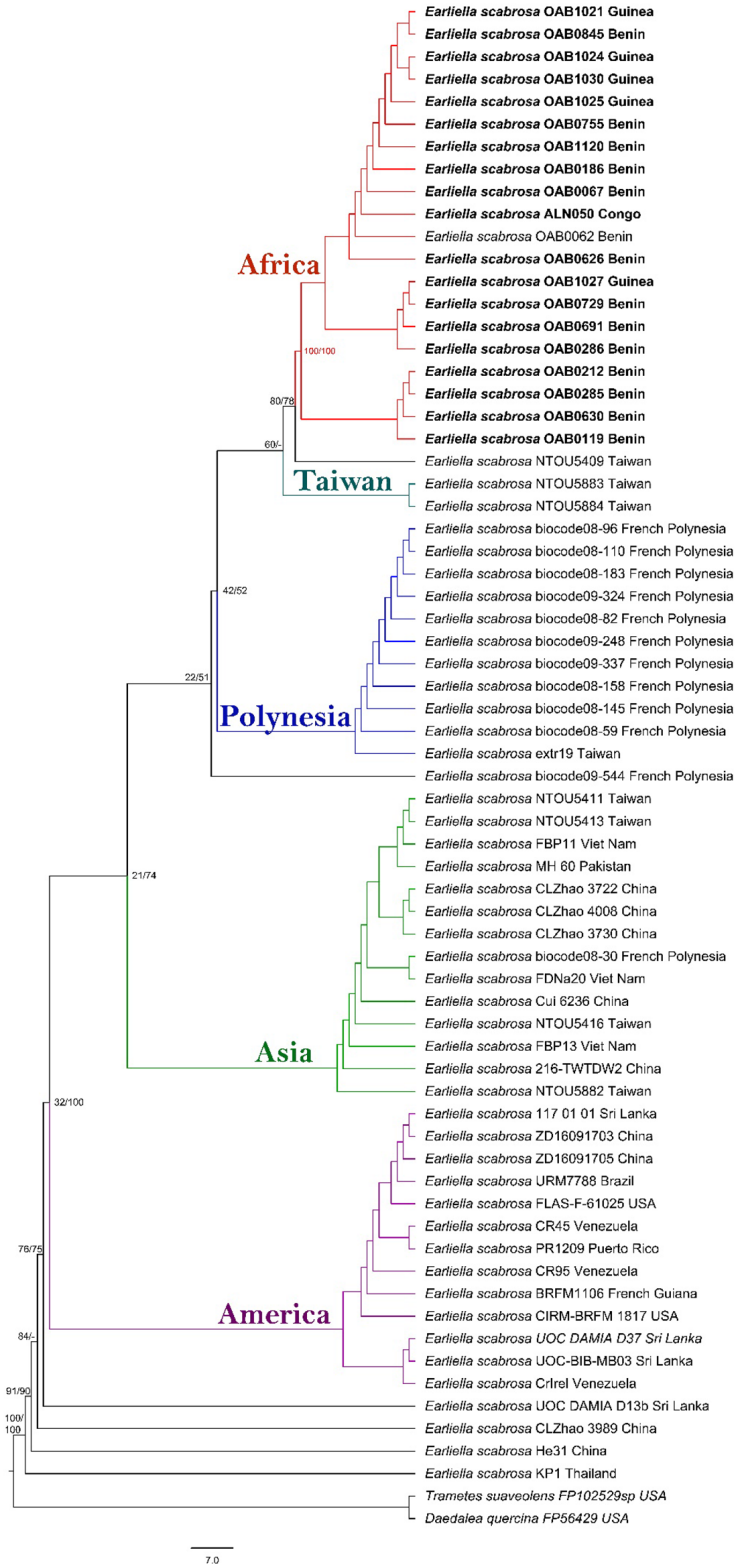
This multilocus ML/BI tree was constructed from the combined alignment of ITS, LSU, and EF1α sequences showing phylogenetic relationships between the 66 collections of *Earliella scabrosa*. The groups highlighted bear the names of the origins of most of the sequences in the respective group.

The alignment of ITS, LSU, and EF1α sequence data of specimens of *E. scabrosa* includes 68 sequences with 2195 characters, 336 distinct patterns, 77 parsimony-informative sites, 301 singleton sites, and 1817 constant sites. The topology of species resulting from ML and BI analyses is congruent. Different groups that reflect populations of *E. scabrosa* with moderate to high support values and evident geographical patterns regarding the origin of collection are highlighted (Fig. 5).

## Discussion

We used the combination of haplotype networks and phylogenetic trees to analyse the genetic diversity and population differentiation within and among samples of *Earliella scabrosa* from its pantropical range of distribution. The phylogenetic tree revealed distinct lineages among the populations of *E*. *scabrosa*. Meanwhile, the haplotype network provided additional quantitative information on how haplotypes are shared between populations of *E*. *scabrosa*.

### Haplotype diversity, population structure and genetic differentiation

In this study, we found a high level of genetic diversity (haplotype diversity, Hd = 0.88) across the known geographical range of *E*. *scabrosa* based on ITS sequences. Similar levels of genetic variability are commonly observed in wild populations with broad ecological niches, and/or wide geographical distribution, such as *Funneliformis mosseae* (T.H. Nicolson & Gerd.) C. Walker & A. Schüßler^63^ and *Schizophyllum commune* Fr.^38,64^. However, the level of genetic diversity detected in our study could be the direct consequence of a single gene used or the nuclear ribosomal region used as other studies on genetic diversity have revealed a difference between the ribosomal and the ISSR markers^7,65^. Here, we were not able to combine both regions or several gene loci because corresponding sequence data are not available in GenBank. Future studies to sequence and combine more genes from different populations of *E*. *scabrosa* will increase our understanding on the genetic diversity of this species.

The 24 haplotypes detected in our analyses are revealed for the first time, and among them, two haplotypes (h20 and h21) are detected from African specimens. Our haplotype analyses reveal a dominance of unique haplotypes (20 out of 24 in total) which are mostly found in Taiwan and French Polynesia. Because our sample sizes are relatively small for several sites, it is possible that more ITS haplotypes might be found if more extensive sampling were conducted and that wider distributions of some of these unique haplotypes might be revealed.

Within the studied populations of *E*. *scabrosa*, haplotypes are hardly shared. Of the 24 haplotypes detected, a few of them (h1, h2, h4, h20) were found distributed in multiple geographical areas (Fig. 3). This result is consistent with a certain degree of gene flow between geographical populations of this species in nature. While evidence for certain degree of gene flow has been found in *E. scabrosa*, our population genetic analyses also suggest that, overall, gene flow was somewhat limited. Previous studies on the gene flow of fungal have shown that gene flow can be short distance or long distance and both of them are generally mediated by wind, human or animal activities^63,66^. In this study, h20 is only shared between African specimens from Congo Republic, Guinea and Benin. The distances between Benin and Guinea on the one hand and Benin and Congo Republic on the other are about 1500 km and 2000 km. Similar spore dispersals (up to 2000 km) were reported in several fungi^67–69^. Thus, we can assume that wind-driven dispersal of basidiospores is probably responsible for the observed gene flow in the African samples. Unlike h20, which is distributed only among samples from the same continent, h1 is shared between America and China, h2 between Sri Lanka and Venezuela, and h4 between Asia and French Polynesia. The geographic distance between these abovementioned populations was much larger than the short dispersal distance of fungal spores. However, even if long-distance gene flow was relatively much rarer^70^, this idea cannot be ruled out as several fungi are known to be influenced by human activity, including plant pathogens^71,72^ and fungi that are found on substrates used by humans like *E. scabrosa*^22^. For example, in natural populations of the button mushroom *Agaricus bisporus* (J.E. Lange) Imbach, the sharing of certain genotypes was found for strains from different regions, countries and even continents^73,74^. Because those genotypes were identical or very similar to cultivated strains, it was suggested that human-aided dispersal was responsible for such long-distance gene flow in *A*. *bisporus*. A similar process could account for the wide distribution of h1, h2, and h4 in our samples.

### Phylogenetics and taxonomy

We detected some level of intraspecific variation within the population of *E*. *scabrosa* even at the fine geographical scale (country level). For example, with an intraspecific divergence of 0–0.2%, populations of *E*. *scabrosa* in China, Venezuela, Taiwan, and French Polynesia might be split into several hypothetical species. However, considering an intraspecific divergence of 0.3%, all sequences of *E*. *scabrosa* sequences are grouped into a single species. This means ITS region may include considerable intraspecific variation, which can lead to oversplitting of species during DNA barcoding analyses^75^. The intraspecific divergence found in this study corresponds well with data available in literature on intraspecific ITS variability of 0–3% in the fungal kingdom^36,76^. Seena et al*.*^77^, when proposing the internal transcribed spacer gene region as a barcode for identifying aquatic hyphomycete species, found also a 0.3% variation within *Articulospora tetracladia* Ingold. The difference in numbers of hypothetical species according to the intraspecific divergence suggests that these hypothetical species should not be used as phylogenetic species, but an extra clustering step is needed to approach species-level resolution. Phylogenetic analyses grouped *E*. *scabrosa* sequences in several groups of which five displaying well evidenced geographical patterns. The phylogenetic patterns within *E*. *scabrosa* can be attributed to geographic hypotheses. Our five well distinct groups showed a preference or tendency for geographic regions. The African group is geographically restricted to specimens from African countries. The Polynesian group is mostly represented by Polynesian samples with an additional collection from Taiwan. Samples forming the Asia group are widely distributed across Asia countries with one collection from French Polynesia. The America group includes American samples as well as multiple samples from China and Sri Lanka. The heterogeneity within some geographical groups confirmed the sharing haplotypes between these populations. Only specimens from the African group are available and examination of these has not revealed any anatomical differences, although distinct morphological characters (presence or absence of reddish cuticles on the basidiomata, the effused basidiomata in some and resupinate in others) have been observed within the specimens of this group. As with the ABGD, the 20 sequences from the African specimens formed a well-defined group and no other sequences from other areas fell into this group. We therefore suspect that geographically separated populations of *E*. *scabrosa* are genetically divergent, but difficult to separate based on morphology. The results from the Mantel tests and linear regression supports the hypothesis that the genetic relationship among populations is closely associated with geographic distance (Table 2, Fig. 4). AMOVA tests also confirmed that geographic separation contributed significantly to the observed sequence variation and approximately 82% of observed differentiations were partitioned among populations. Similar types of geographic patterns of DNA sequence variation have been observed in many other fungal groups. For example, distinct alleles were observed in geographically separated populations of *Russula brevipes* Peck^78,79^. Each group identified here and especially the African one could become a distinct phylogenetic species knowing that long-term geographical isolation could favour the process of allopatric divergence of different populations^80^.

## Conclusion

This study revealed five distinct groups within *E*. *scabrosa* and a correlation between genetic and geographic distances. The intraspecific divergences (0–0.3%), however, are relatively low, so cryptic taxa apparently are not present. The different groups may eventually evolve into phylogenetically distinct species in the long term, as geographical isolation could favour the process of allopatric divergence. More extensive sampling and analyses combining several makers may reveal additional distinct lineages as well as novel distribution patterns within this species in its geographical areas of distribution.

### Supplementary Information

Supplementary Information.

## Data Availability

Newly generated sequences are available in GenBank and the accession numbers are given in Table1. Alignment, phylogenetic tree, and accession numbers of newly generated sequences will be public after the paper is published. Collected specimens are available at the mycological herbaria of the University of Parakou (UNIPAR) in Benin.
