# Supplementary material for: Genetic diversity and population differentiation in Earliella scabrosa, a pantropical species of Polyporales
Source: Sci Rep. 2023 Dec 27;13:23020. doi: 10.1038/s41598-023-50398-5 (PMC10754928; doi:10.1038/s41598-023-50398-5)
Supplement: Supplementary file 1 — Supplementary Information. [file 41598_2023_50398_MOESM1_ESM.pdf]

Supplementary S1. ML tree was constructed from the ITS sequences showing the phylogenetic relationships between the 95 Earliella scabrosa collections. Sequences outside the true Earliella scabrosa are highlighted in red and are not taken into account in the rest of the analyses.

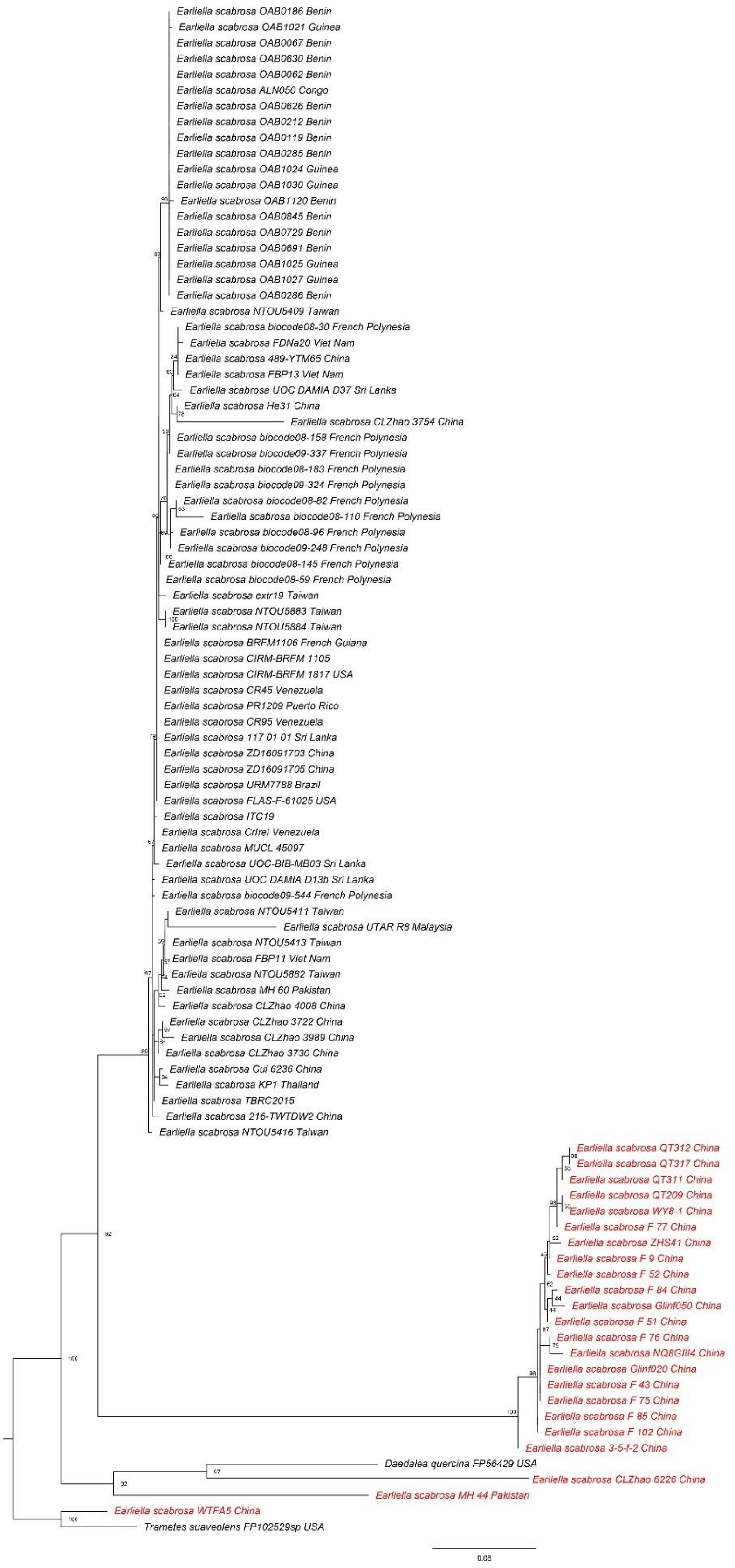

Supplementary S2. Data on the Earliella scabrosa collections used in this study

Species name	Vouchers	Forest type	Country
Earliella scabrosa	OAB0062	Semi decidious Dense forest	Benin
Earliella scabrosa	OAB0067	Semi decidious Dense forest	Benin
Earliella scabrosa	OAB0119	Woodland	Benin
Earliella scabrosa	OAB0186	Woodland	Benin
Earliella scabrosa	OAB0212	Savanna	Benin
Earliella scabrosa	OAB0285	Semi decidious Dense forest	Benin
Earliella scabrosa	OAB0286	Semi decidious Dense forest	Benin
Earliella scabrosa	OAB0691	Dense dry forest	Benin
Earliella scabrosa	OAB0626	Dense dry forest	Benin
Earliella scabrosa	OAB0630	Dense dry forest	Benin
Earliella scabrosa	OAB0729	Semi decidious Dense forest	Benin
Earliella scabrosa	OAB0755	Woodland	Benin
Earliella scabrosa	OAB0845	Woodland	Benin
Earliella scabrosa	OAB1120	Dense dry forest	Benin
Earliella scabrosa	OAB1021	Woodland Serekoro	Guinea
Earliella scabrosa	OAB1024	Woodland Serekoro	Guinea
Earliella scabrosa	OAB1025	Woodland Serekoro	Guinea
Earliella scabrosa	OAB1027	Woodland Kankan	Guinea
Earliella scabrosa	OAB1030	Woodland Kankan	Guinea
Earliella scabrosa	ALN050	Mbindjo forest	Congo

Supplementary S3. Result from species delimitation analysis using the Automated Barcode Gap Discovery (ABGD)

Group [1] n: 10 ;id: Earliella scabrosa 117 01 01 Sri Lanka; Earliella scabrosa CIRM-BRFM 1817 USA; Earliella scabrosa CR45 Venezuela; Earliella scabrosa CR95 Venezuela; Earliella scabrosa PR1209 Puerto Rico; Earliella scabrosa FLAS-F-61025 USA; Earliella scabrosa URM7788 Brazil; Earliella scabrosa ZD16091703 China; Earliella scabrosa ZD16091705 China; Earliella scabrosa BRFM1106 French Guiana
Group [2] n: 2 ;id: Earliella scabrosa CrIrel Venezuela; Earliella scabrosa UOC DAMIA D37 Sri Lanka
Group[3] n: 17 ;id: Earliella scabrosa biocode08-59 French Polynesia; Earliella scabrosa biocode08-30 French Polynesia; Earliella scabrosa FBP13 Viet Nam; Earliella scabrosa biocode08-82 French Polynesia; Earliella scabrosa biocode08-183 French Polynesia; Earliella scabrosa biocode09-337 French Polynesia; Earliella scabrosa biocode08-158 French Polynesia; Earliella scabrosa biocode08-145 French Polynesia; Earliella scabrosa biocode09-248 French Polynesia; Earliella scabrosa biocode09-324 French Polynesia ; Earliella scabrosa CLZhao 3730 China; Earliella scabrosa NTOU5411 Taiwan; Earliella scabrosa MH 60 Pakistan; Earliella scabrosa FBP11 Viet Nam; Earliella scabrosa NTOU5413 Taiwan; Earliella scabrosa NTOU5882 Taiwan; Earliella scabrosa biocode08-110 French Polynesia
Group [4] n: 1 ;id: Earliella scabrosa UOC DAMIA D13b Sri Lanka
Group [5] n: 1 ;id: Earliella scabrosa extr19 Taiwan
Group [6] n: 1 ;id: Earliella scabrosa biocode09-544 French Polynesia
Group [7] n: 1 ;id: Earliella scabrosa biocode08-96 French Polynesia
Group [8] n: 1 ;id: Earliella scabrosa He31 China
Group [9] n: 3 ;id: Earliella scabrosa CLZhao 3722 China; Earliella scabrosa CLZhao 3989 China; Earliella scabrosa CLZhao 4008 China
Group [10] n: 1 ;id: Earliella scabrosa Cui 6236 China
Group [11] n: 1 ;id: Earliella scabrosa KP1 Thailand
Group [12] n: 1 ;id: Earliella scabrosa UOC-BIB-MB03 Sri Lanka
Group [13] n: 1 ;id: Earliella scabrosa FDNa20 Viet Nam
Group [14] n: 1 ;id: Earliella scabrosa 216-TWTDW2 China
Group [15] n: 1 ;id: Earliella scabrosa NTOU5416 Taiwan
Group [16] n: 1 ;id: Earliella scabrosa NTOU5409 Taiwan
Group [17] n: 20 ;id: Earliella scabrosa OAB0691 Benin; Earliella scabrosa OAB0119 Benin; Earliella scabrosa OAB0212 Benin; Earliella scabrosa OAB0286 Benin; Earliella scabrosa ALN050 Congo; Earliella scabrosa OAB0067 Benin; Earliella scabrosa OAB0186 Benin; Earliella scabrosa OAB0285 Benin; Earliella scabrosa OAB0626 Benin; Earliella scabrosa OAB1027 Guinea; Earliella scabrosa OAB1021 Guinea; Earliella scabrosa OAB1024 Guinea; Earliella scabrosa OAB1025 Guinea; Earliella scabrosa OAB1030 Guinea; Earliella scabrosa OAB0062 Benin; Earliella scabrosa OAB0755 Benin; Earliella scabrosa OAB0630 Benin; Earliella scabrosa OAB1120 Benin; Earliella scabrosa OAB0729 Benin; Earliella scabrosa OAB0845 Benin
Group [18] n: 2 ;id: Earliella scabrosa NTOU5883 Taiwan; Earliella scabrosa NTOU5884 Taiwan

Supplementary S4. Result from species delimitation analysis using the Automated Barcode Gap Discovery (ABGD)

Group[1] n: 66 ;id: Earliella scabrosa 117 01 01 Sri Lanka; Earliella scabrosa CIRM-BRFM 1817 USA; Earliella scabrosa CR45 Venezuela; Earliella scabrosa CR95 Venezuela; Earliella scabrosa PR1209 Puerto Rico; Earliella scabrosa FLAS-F-61025 USA; Earliella scabrosa CrIrel Venezuela; Earliella scabrosa URM7788 Brazil; Earliella scabrosa ZD16091703 China; Earliella scabrosa ZD16091705 China; Earliella scabrosa biocode08-59 French Polynesia; Earliella scabrosa biocode08-30 French Polynesia; Earliella scabrosa FBP13 Viet Nam; Earliella scabrosa UOC DAMIA D13b Sri Lanka; Earliella scabrosa BRFM1106 French Guiana; Earliella scabrosa UOC DAMIA D37 Sri Lanka; Earliella scabrosa biocode08-82 French Polynesia; Earliella scabrosa biocode08-183 French Polynesia; Earliella scabrosa biocode09-337 French Polynesia; Earliella scabrosa biocode08-158 French Polynesia; Earliella scabrosa biocode08-145 French Polynesia; Earliella scabrosa extr19 Taiwan; Earliella scabrosa biocode09-544 French Polynesia; Earliella scabrosa biocode08-96 French Polynesia; Earliella scabrosa biocode09-248 French Polynesia; Earliella scabrosa biocode09-324 French Polynesia; Earliella scabrosa He31 China; Earliella scabrosa CLZhao 3722 China; Earliella scabrosa CLZhao 3730 China; Earliella scabrosa NTOU5411 Taiwan; Earliella scabrosa CLZhao 3989 China; Earliella scabrosa Cui 6236 China; Earliella scabrosa MH 60 Pakistan; Earliella scabrosa KP1 Thailand; Earliella scabrosa FBP11 Viet Nam; Earliella scabrosa UOC-BIB-MB03 Sri Lanka; Earliella scabrosa FDNa20 Viet Nam; Earliella scabrosa 216-TWTDW2 China; Earliella scabrosa NTOU5416 Taiwan; Earliella scabrosa NTOU5409 Taiwan; Earliella scabrosa NTOU5413 Taiwan; Earliella scabrosa CLZhao 4008 China; Earliella scabrosa OAB0691 Benin Earliella scabrosa OAB0119 Benin; Earliella scabrosa OAB0212 Benin; Earliella scabrosa OAB0286 Benin; Earliella scabrosa ALN050 Congo; Earliella scabrosa OAB0067 Benin; Earliella scabrosa OAB0186 Benin; Earliella scabrosa OAB0285 Benin; Earliella scabrosa OAB0626 Benin; Earliella scabrosa OAB1027 Guinea; Earliella scabrosa OAB1021 Guinea; Earliella scabrosa OAB1024 Guinea; Earliella scabrosa OAB1025 Guinea; Earliella scabrosa OAB1030 Guinea; Earliella scabrosa OAB0062 Benin; Earliella scabrosa OAB0755 Benin; Earliella scabrosa OAB0630 Benin; Earliella scabrosa OAB1120 Benin; Earliella scabrosa OAB0729 Benin; Earliella scabrosa OAB0845 Benin; Earliella scabrosa NTOU5882 Taiwan; Earliella scabrosa NTOU5883 Taiwan; Earliella scabrosa NTOU5884 Taiwan; Earliella scabrosa biocode08-110 French Polynesia
